# Two new families with hereditary minimal change disease

**DOI:** 10.1186/1471-2369-14-65

**Published:** 2013-03-22

**Authors:** Hassib Chehade, Francois Cachat, Eric Girardin, Samuel Rotman, Antonio Jorge Correia, Florence Fellmann, Olivier Bonny

**Affiliations:** 1 Division of Pediatric Nephrology of West Switzerland, Lausanne University Hospital, Lausanne, Switzerland; 2 Department of Pathology, Lausanne University Hospital, Lausanne, Switzerland; 3 Pediatric Nephrology, Children’s Hospital Coimbra, Coimbra, Portugal; 4 Service of Medical Genetics, Lausanne University Hospital, Lausanne, Switzerland; 5 Service of Nephrology, Lausanne University Hospital, Lausanne, Switzerland

**Keywords:** Nephrotic syndrome, Minimal change disease, Heredity, Genetics, Steroids

## Abstract

**Background:**

Steroid-sensitive idiopathic nephrotic syndrome (SSINS) is most often encountered in sporadic cases of minimal change disease (MCD). Only rare cases of familial forms of MCD have been reported and most of them only in one generation. The scarcity of data has precluded unraveling the underlying genetic defect and candidate gene approaches have been unsuccessful. Here we report two families with related SSINS cases and review the related literature.

**Case presentation:**

Two siblings and a cousin (first family), and a father and his son (second family), are reported with SSINS due to MCD. Patients have been followed up for more than 12 years and a renal biopsy was performed in three cases, demonstrating typical features of MCD. The course of the disease was remarkable because of several relapses treated with steroids. In three cases, mycophenolate mofetil or cyclosporine was added.

**Conclusion:**

Familial SSINS due to MCD is extremely rare and no genetic defect has been identified so far. Reporting cases of hereditary MCD will allow further genetic studies which will ultimately help unravel the molecular basis of this disease.

## Background

Idiopathic nephrotic syndrome (INS) in children is caused by various entities that differ in their histopathological forms and their clinical course [1]. Minimal change disease (MCD) and focal segmental glomerulosclerosis (FSGS) are the most common causes of INS representing 80% and 20% of the cases respectively [1]. The clinical outcome of INS is determined by the responsiveness to treatment by steroids. Most steroid-sensitive INS (SSINS) are due to MCD, while steroid-resistant INS (SRINS) are mostly represented by FSGS.

Although INS is well known as a sporadic disease, familial occurrences with autosomal dominant or recessive mode of inheritance have been described especially in FSGS forms [2]. Several genes have been associated with or shown to be causative for some specific forms of INS, mostly steroid-resistant, including *NPHS1*, *NPHS2, PLCE1, WT1*, *ACTN4*, *TRPC6, CD2AP, APOL1* or *INF2*[3]. However, reports of familial MCD are scarce and no causal gene has been identified yet. Here, we describe five cases of steroid sensitive MCD in two non-consanguineous families and perform a review of the literature. The objective of our report is to encourage physicians to identify and characterize genetic causes of MCD. This may help to understand more precisely the pathophysiological mechanisms of INS and provide a first step toward the identification of the underlying genetic cause.

## Cases presentation

### Family 1

**Figure 1:**
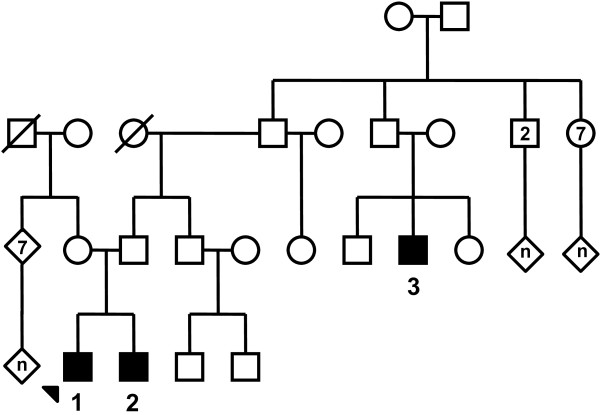
**Extensive pedigree of the first family. **Three members of this Portuguese non-consanguineous family were affected by steroid-sensitive nephrotic syndrome, type minimal change disease: two affected siblings (cases **1 **and **2**) and the first cousin once removed (case **3**). The index case (case **1**) is indicated by an arrow head. The numbers inside figures indicate the number of males (square), females (rounds) or non-specified sex (lozenges) of the family.

We describe a Portuguese non-consanguineous family (see pedigree: Figure 1) in which two siblings (cases 1 and 2) and one cousin (case 3) were diagnosed with MCD INS.

### Case 1

A 6 year old boy, with no previous medical history, presented with fatigue and facial edema. Physical examination showed moderate periorbital edema. Blood pressure was within normal range for age (105/69 mmHg). Heart sounds and lung examination were unremarkable. Abdomen was soft, not distended, and no mass, shifting dullness or hepatosplenomegaly were found. He had normal male genitalia with mild scrotal edema. The dorsal surfaces of hands and feet had mild pitting edema. Urine analysis by dipstick showed 4+ proteinuria with no hematuria. Urine spot showed a nephrotic range proteinuria (protein/creatinine ratio of 2000 g/mol). The blood chemistry panel was remarkable for plasma protein level of 35 g/l and serum albumin of 10 g/l. BUN and creatinine levels were normal, and no electrolyte disturbance was noted. The diagnosis of INS was posed and the patient was treated with oral prednisone (60 mg/m^2^/day b.i.d.). Feet edema and proteinuria gradually resolved over the course of treatment. He was followed up as outpatient and did monitor daily albuminuria with urine dipsticks. Corticosteroids were tapered off progressively and stopped. Three months later, the patient relapsed after a minor respiratory infection with re-appearance of proteinuria. Treatment with steroids was re-initiated for two months. Eighteen months later, the patient remains compensated without steroids and has normal blood pressure and normal renal function.

### Case 2

**Figure 2:**
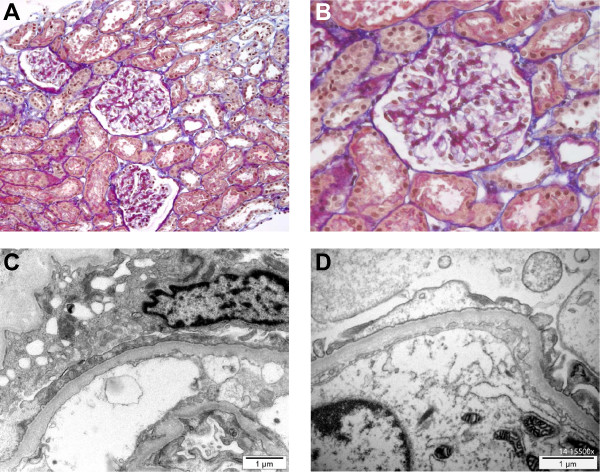
**Renal biopsy of case 2 showing normal morphology by light microscopy (H&E staining, panel A, magnification 100×, panel B, magnification 400×).** Electron microscopy reveals podocyte foot effacement without any other structural abnormality (panel **C**, magnification 11500× and panel **D**, magnification 15500×).

The brother of case 1, a child with no previous medical history, presented at 3 years of age with mild facial edema without any other clinical sign. Blood pressure was normal. Laboratory tests showed proteinuria of nephrotic range (protein/creatinine ratio 750 g/mol). Blood chemistry showed low levels of total protein (45 g/l) and serum albumin (18 g/l), but BUN and creatinine concentrations were normal. INS was diagnosed and the infant was treated with oral prednisone 60 mg/m^2^/day b.i.d. He was followed up in the outpatient clinic and was monitoring proteinuria with dipsticks every day. Edema and proteinuria gradually resolved under treatment and steroids were tapered off with an initial favorable course. However, 2 months after the interruption of the corticosteroids treatment, the patient presented several relapses, all steroid-sensitive and every time triggered by respiratory or gastro-intestinal viral infections. A renal biopsy was performed and showed all the typical features of MCD (Figure 2), but no sign of FSGS. Immunofluorescence staining was negative. The patient was treated with mycophenolate mofetil (MMF) in addition to steroids with favorable outcome. Steroids were then progressively tapered down and stopped.

Asking for family history of the two siblings lead to identification of a first cousin once removed with a history of INS due to MCD (Figure 1). Of note, family history was unremarkable for chronic kidney failure or renal graft.

### Case 3

**Figure 3:**
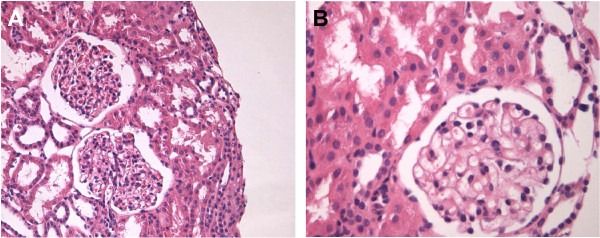
**Renal biopsy of case 3. **Normal light microscopy (H&E staining, panel **A**, magnification 200×, panel **B**, magnification 400×).

This now 14 year old boy was initially diagnosed with nephrotic syndrome at the age of 2 and was successfully treated with corticosteroids. He suffered from several relapses and was treated with oral cyclophosphamide. A renal biopsy at age 3 shows normal morphology at light microscopy, in particular no glomerulosclerosis and interstitial fibrosis (Figure 3). No electronic microscopy has been available. Another round of cyclophosphamide was given at age 4 due to several relapses. At age 4.5, the child presented another relapse which was treated with low dose corticosteroids and cyclosporine was introduced for a 12 month period. At age 8, another relapse was treated with a full dose of corticosteroids (60 mg/m^2^/day) with favorable response and was maintained afterwards at low dose on alternate days. A treatment by mycophenolate mofetil (600 mg/m^2^/day) was introduced. The course of the disease was since favorable with rarer relapse episodes. At the last follow up (age 14), physical exam was normal with blood pressure of 111/65 mmHg and serum creatinine level was in the normal range.

### Family 2

**Figure 4:**
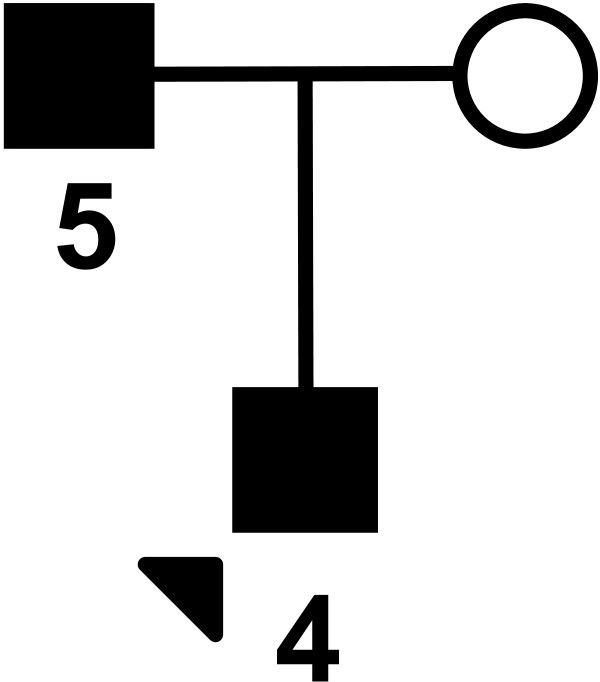
**Pedigree of the second family. **The index case is indicated by an arrow head.

Here we describe a French non-consanguineous family (see pedigree: Figure 4) in which a father and his son (cases 4 and 5) were diagnosed with MCD INS.

### Case 4

**Figure 5:**
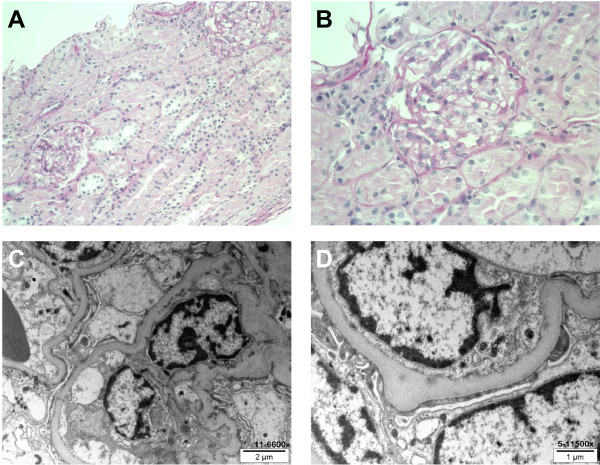
**Renal biopsy of case 4 shows normal light microscopy (H&E staining, panel A, magnification 200×, panel B, magnification 400×). **Electron microscopy shows podocyte foot fusion (panel **C**, magnification 6600× and panel **D**, magnification D, 11500×).

A 4 year old boy, with no previous medical history, presented with periorbital edema. Blood pressure was within normal range for age (100/59 mmHg). He had mild edema of the dorsal surfaces of hands, feet and mild scrotal edema. Urine analysis by dipstick showed 4+ proteinuria with no hematuria. Urine spot showed a nephrotic range proteinuria (protein/creatinine ratio of 2450 g/mol). The blood chemistry panel was remarkable for plasma protein level of 39 g/l and serum albumin of 12 g/l. BUN, creatinine levels were normal and no electrolyte disturbance was noted. The diagnosis of INS was posed and the patient was treated with oral prednisone (60 mg/m^2^/day b.i.d.). Proteinuria resolved after 7 days of treatment and peripheral edema gradually disappeared over the course of treatment. He was followed up as outpatient and did monitor daily albuminuria with urine dipsticks. Corticosteroids were tapered down progressively for 6 months and stopped. Two months later, the patient presented a relapse possibly induced by a respiratory infection. Treatment with steroids was re-initiated at 60 mg/m^2^/day and subsequently decreased, but at the doses of 20 mg/m^2^/day, proteinuria relapsed. A biopsy was performed showing all the features typical for MCD (Figure 5), with no sign of FSGS. Immunofluorescence staining was negative. Prednisone treatment was increased to 60 mg/m^2^/day and mycophenolate mofetil was added with a favorable outcome on proteinuria. Steroids were then progressively tapered down and stopped. Three months later, the patient remains compensated without steroids, has normal blood pressure and normal renal function.

### Case 5

The 37 year old father of case 4 was hospitalized at the age of 10 for peripheral edema, nephrotic range proteinuria and hypoalbuminemia without renal failure or arterial hypertension. The diagnosis of nephrotic syndrome was made and treated with prednisone that was tapered off progressively over 6 months. The evolution was then favorable without relapses. At present time, the patient displays normal renal function, normal blood pressure of 120/78 mmHg and no proteinuria (protein/creatinine ratio: 9 g/mol).

## Conclusion

In the past years, many familial FSGS cases have been reported and genetic studies have identified mutations in several genes coding for proteins of the slit diaphragm complex and the podocyte which leads to autosomal recessive (*NPHS1, NPHS2*) or autosomal dominant (ACTN4, CD2AP, TRPC6 genes) steroid-resistant FSGS [1,4-6]. Identification of genes related to FSGS has contributed significantly to a better understanding of the molecular paths involved in SRINS and is an important determinant for the course of the disease. For instance, relapses in renal transplant recipient carrying FSGS genes mutations are rare as compared to FSGS kidney transplant recipients without any gene mutation [3]. But while important genetic clues have been identified for familial steroid-resistant INS and FSGS, reports of genes causative for familial steroid sensitive INS and MCD are still lacking. This might be due to the low prevalence of the disease and to the few numbers of cases described so far. Here, we report two novel families with 5 cases of MCD. We encourage clinicians to report their cases in order to collect enough families to conduct genetic studies.

Literature review (using PubMed Advanced Search Builder, date: 1960–1980 with the following key words: familial nephrotic syndrome, and the date: 1980–2011 using the following key words: familial minimal change disease, familial nephrotic syndrome) of familial cases of SSINS revealed several reported cases within siblings (Table 1) and only sixteen families with SSINS affecting two generations (Table 2). Several interesting features taken from these reports may help in managing these cases. In their report of fifteen families with childhood-onset SSINS, Fuchshuber et al. [7] reported that the clinical course of the familial forms was equivalent to sporadic SSINS cases. A strong heritability of the age of onset of the disease was suggested. In this first large report of familial SSINS, linkage with the candidate gene NPHS2 was excluded and the authors concluded the existence of a distinct gene locus for familial SSINS. Landau et al. [8] reported on several extensive Bedouin families affected by SSINS with similar clinical course - in terms of age of onset, male predominance and spontaneous cure at puberty -compared to those in sporadic cases. By linkage analysis, the authors showed a complete absence of linkage with the usual candidate genes loci implicated in nephrotic syndrome or other glomerulopathies and they advised for more specific genome-wide screening with a denser marker set. In three families with SSINS, Ruf et al. [9] were able to pinpoint a locus on chromosome 2p12-p13.2, and also demonstrated clear evidence for genetic locus heterogeneity upon examination of ten additional families with SSINS. The rare cases of familial SSINS reported in the literature confirm that the disease course is similar to sporadic cases of SSINS, but clearly distinct from familial FSGS nephrotic syndrome.

**Table 1:** Reported siblings with SSINS

**References**	**Year**	**Familial cases with SSINS**	**Histopathological confirmation of MCD**
Roy S et al. [10]	1971	Identical twins	Biopsy performed in 2 cases
Moncrieff MW et al. [11]	1973	18 cases in 9 families	Biopsy performed in 12 cases
White RH et al. [12]	1973	12 cases from 24 centers in Europe	Biopsy performed in 12 cases
Bader BI et al. [13]	1974	1 affected sibling pairs	Biopsy performed in 1 case
McEnery PT et al. [14]	1989	2 cases in a family	Data not available
Awadalla NB et al. [15]	1989	3 cases in a family	Biopsy performed in 3 cases
Fuchshuber A et al. [7]	2001	32 cases in 15 families	Biopsy performed in 12 cases
Ruf RG et al. [9]	2003	7 cases in 3 families	Biopsy performed in 2 cases
Landau D et al. [8]	2007	6 cases in 2 related families	No biopsy performed
Roberts IS et al. [16]	2008	2 cases in a family	Biopsy performed in one case
Motoyama O et al. [17]	2009	2 cases in a family	No biopsy performed

**Table 2:** Reported familial cases with SSINS in two generations

**References**	**Year**	**Familial cases with SSINS in two generations**	**Histopathological confirmation of MCD**
White RH et al. [12]	1973	A father and his daughter	Biopsy of the daughter only
Bader BI et al. [13]	1974	2 affected first cousins from a consanguineous family	Biopsy performed in both cases
McEnery PT et al. [14]	1989	A father and his son	
		Two families with 2 affected first cousins	Data not available
Awadalla NB et al. [15]	1989	3 cases in a family and a cousin	Biopsy performed in all 3 cases
Landau D et al. [8]	2007	- 2 families with parent/child affected	Biopsy performed in one case of the 14 affected Bedouin consanguineous family members
		- 2 Bedouin consanguineous family with 14 affected members	
		- 5 non-related Bedouin families with 10 affected members	
Motoyama O et al. [17]	2009	A father and a daughter	No biopsy performed

Cases of familial SSINS spread over two generations have rarely been described (see list in Table 2). Outcome, in terms of renal function and blood pressure, is usually favorable [7] compared to familial FSGS [18-20].

As the majority of familial cases of SSINS reported in the literature is limited to one generation of siblings (Table 1), the first genetic inheritance pattern suggested was autosomal recessive or a possible germinal mosaicism. However, description of familial SSINS cases in two generations (Table 2) with transmission from father to children broadens the disease inheritance possibilities to autosomal dominant transmission model with variable penetrance. Altogether, analysis of the data issued from the literature does not allow definitive conclusions about the inheritance pattern of familial MCD and is permissive for different possible transmission hypothesis, including autosomal recessive, autosomal dominant with variable penetrance or genetic heterogeneity. In addition, a more complex inheritance pattern associated with oligogenic predisposition and possible environmental effects is also possible. More reports of familial MCD are needed in order to understand the disease transmission pattern.

In this report, case 1 presented with typical INS at age six and the follow-up was marked by a single relapse occurring three months after the interruption of the steroids. Renal biopsy was not performed in that case due to rapid favorable outcome. The second case presented with INS at the age of 3 with an initial favorable disease course, later complicated by frequent relapses. A renal biopsy confirmed the diagnosis of MCD. The child eventually showed a favorable evolution after the introduction of MMF. Case 3, a first cousin once removed, presented with a classical INS at age 6 and the renal biopsy showed typical MCD. Despite treatment with corticosteroids, frequent relapses were observed and treated with cyclophosphamide, cyclosporine, and finally, MMF. These 3 cases had normal renal function (estimated GFR using the revised Schwartz formula were 96, 97 and 99 ml/min 1.73 m^2^ respectively) and blood pressure. Pedigree of this family is compatible with an autosomal dominant inheritance with variable penetrance, but other forms of heritability are possible. A de novo mutation in this family of two affected siblings and their cousin seems less probable though. In the second reported family, renal outcome was also favorable and the pedigree is compatible with an autosomal dominant inheritance. Overall, all familial cases of MCD reported here had a clinical presentation in terms of age of onset, symptoms during the initial phase, renal morphology and outcome close to other familial cases described in the literature.

We are aware of this report’s few limitations. First, it is descriptive and does not propose precise genetic or molecular mechanisms that could explain familial MCD. However, this publication is meant to encourage further reports of similar rare cases that, once collected, may allow wider genetic analysis. Second, recent reports have suggested a role of CD80 induction in the occurrence of *sporadic* MCD [21]. We did not dose soluble CD80 in the urine of the patients presented here and therefore could not conclude about the possible value of this biomarker and this proposed pathophysiological mechanism for *familial* MCD.

In summary, here we describe five cases issued from two families with steroid sensitive INS occurring in two generations. The clinical course of these cases was similar to sporadic INS regarding the age of onset, clinical presentation and the presence of minor infection prior to the onset of recurrences, response to treatment and disease outcome. This confirms the few previous observations of familial MCD reported in the literature. The aim of this paper is to emphasize the importance of identifying these families in order to allow further genetic analysis, determine mode of inheritance and understand the mechanisms of INS appearance.

## Consent

Written informed consent was obtained from the patient for publication of this Case report and any accompanying images. A copy of the written consent is available for review by the Editor of this journal.

## Competing interest

No part of this manuscript has been previously published. The authors declare they have no conflict of interest related to this manuscript.

## Authors’ contributions

HC, FC, EG, AJC treated and followed the patients. SR provided the histopathological analysis. FF provided genetic counsels. OB and HC prepared the manuscript. All authors read and approved the final manuscript.

## Pre-publication history

The pre-publication history for this paper can be accessed here:

http://www.biomedcentral.com/1471-2369/14/65/prepub
